# Effects of Proteinuria on Cerebral and Muscle Oxygenation and Microvascular Reactivity in Patients With Pre-Dialysis Chronic Kidney Disease: A Post-Hoc Analysis

**DOI:** 10.7759/cureus.84913

**Published:** 2025-05-27

**Authors:** Marieta Theodorakopoulou, Fotini Iatridi, Danai Faitatzidou, Artemios G Karagiannidis, Aggelos Koutlas, Konstantina Dipla, Andreas Zafeiridis, Pantelis Sarafidis

**Affiliations:** 1 First Department of Nephrology, Hippokration Hospital, Aristotle University of Thessaloniki, Thessaloniki, GRC; 2 Department of Sports Science at Serres, Exercise Physiology and Biochemistry Laboratory, Aristotle University of Thessaloniki, Serres, GRC

**Keywords:** cerebral oxygenation, chronic kidney disase (ckd), chronic kidney disease (ckd), muscle oxygenation, near-infrared spectroscopy, proteinuria

## Abstract

Introduction

Vascular dysfunction is a hallmark of chronic kidney disease (CKD), with previous studies showing progressively deteriorating microvascular reactivity in skeletal muscles with advancing CKD stages. Additionally, cognitive impairment is quite common in CKD patients, as significant determinants of brain activation, i.e., cerebral perfusion and oxygenation, are significantly impaired. The aim of this analysis was to investigate, for the first time, the effects of proteinuria on skeletal muscle and cerebral oxygenation, as well as on muscle microvascular reactivity in patients with pre-dialysis CKD.

Methods

A total of 56 patients with pre-dialysis CKD were included in this post-hoc, exploratory analysis and stratified based on the presence of proteinuria (urinary protein-to-creatinine ratio > 200 mg/g). Patients with and without proteinuria were matched in a 1:1 ratio for age, sex, and estimated glomerular filtration rate (eGFR). Near-infrared spectroscopy was used to measure muscle and cerebral oxygenation at rest and after stimulation (occlusion-reperfusion and handgrip exercise).

Results

The two groups were similar in terms of age, eGFR, body mass index, and major comorbidities. Muscle oxygenation did not differ between study groups at rest and during occlusion; however, proteinuric CKD patients presented a trend toward lower tissue saturation index (TSI) during reperfusion (10-second slope: 1.36±0.69 vs. 1.67±0.83, p=0.143) and hyperemic response (7.26±3.98 vs. 8.47±4.52, p=0.296). Regarding cerebral oxygenation, proteinuric patients displayed lower average response during exercise (oxygenated hemoglobin [O_2_Hb]: 0.92±0.78 vs. 1.49±0.86, p=0.012; hemoglobin difference [Hb_diff_]: 1.41±0.96 vs. 1.98±1.12; p=0.044). The average response in total hemoglobin (tHb) (an index of regional blood volume) was also lower (0.43±0.98 vs. 1.00±0.85, p=0.023), but no between-group differences in deoxygenated hemoglobin (HHb) (an index of oxygen extraction capacity) were observed.

Conclusions

In this exploratory analysis, CKD patients with proteinuria showed signs of attenuated cerebral oxygenation during a mild physical task and a possible trend toward modest impairments in skeletal muscle oxidative capacity and microvascular reactivity.

## Introduction

Chronic kidney disease (CKD) is associated with a high burden of morbidity and mortality globally and represents a major source of disability and poor quality of life (QoL) [1]. Exercise intolerance and neurocognitive disorders play a major role in reduced QoL in patients with CKD, contributing to a vicious cycle of inactivity, deconditioning, social isolation, and depression [2]. Prior studies have shown that reduced physical activity is associated with cognitive impairment in CKD and that both exercise intolerance and cognitive deficits appear early in the disease course, worsening progressively toward advanced CKD stages [3,4].

The brain may represent the key link in the strong association between exercise capacity and cognitive performance. Cerebral tissue requires a continuous supply of oxygen to support its high metabolic demands, particularly during periods of increased neural activity, such as physical or cognitive tasks [5]. As neuronal activity increases, oxygen consumption rises, and cerebral blood flow must increase to match this demand. Consequently, even subtle reductions in cerebral perfusion or oxygenation can impair brain function, contributing to both cognitive decline and reduced exercise tolerance [5].

Endothelial and microvascular dysfunction are hallmarks of CKD and play a key role in limiting both cerebral and skeletal muscle perfusion. Endothelial dysfunction occurs early in CKD and shows a progressive impairment with advancing CKD [6,7]. In parallel, it has been associated with target-organ damage, progression of CKD, cardiovascular events, and mortality in these subjects [6,8,9]. Proteinuria, a key marker of glomerular injury, is strongly and independently linked to endothelial dysfunction; more importantly, proteinuria is associated with a heightened risk of adverse cardiovascular and renal outcomes, as well as worse cognitive function and exercise capacity [4,10-12].

Near-infrared spectroscopy (NIRS) is a validated, non-invasive method that provides real-time assessment of local tissue oxygenation and blood flow in both muscle and cerebral tissue [13]. Previous studies have shown that both muscle and cerebral oxygenation assessed with NIRS are impaired in CKD and deteriorate with advancing CKD stages [7,14]. However, it remains unclear whether the presence of proteinuria itself is associated with additional impairment in tissue oxygenation or microvascular responses in CKD.

Therefore, the aim of this analysis was to investigate, for the first time, the association between proteinuria and alterations in skeletal muscle and cerebral oxygenation, as well as impaired muscle microvascular reactivity, in patients with pre-dialysis CKD.

## Materials and methods

Study population

This post-hoc, exploratory analysis includes 56 patients with pre-dialysis CKD (stages 2-4) followed at the outpatient clinic of the First Department of Nephrology, Hippokration Hospital, Aristotle University of Thessaloniki, Thessaloniki, Greece [7]. The study protocol (NCT05250167) was reviewed and approved by the Ethics Committee of the School of Medicine, Aristotle University of Thessaloniki, Thessaloniki, Greece (approval number 4294), and the Institutional Review Board of Hippokration Hospital (issued number 437/17.02.2022). All procedures were performed according to the Declaration of Helsinki (2013 Amendment), and all participants provided informed written consent prior to study enrollment.

Inclusion criteria were as follows: i) age >18 years; ii) patients with CKD at stages 2-4, i.e., with an estimated glomerular filtration rate (eGFR) (using the CKD Epidemiology Collaboration [CKD-EPI] equation) <90 and ≥15 mL/min/1.73m^2^; and iii) provision of informed written consent (Table 1). We excluded patients with i) kidney transplantation, ii) myocardial infarction or unstable angina in the past three months and/or congestive heart failure class III-IV, according to the New York Heart Association criteria, iii) history of stroke, dementia, or other severe neurologic disorders (e.g., Parkinson's disease, multiple sclerosis, etc.), iv) severe mental disorders, v) muscle disorders, vi) history of drug or alcohol abuse, vii) active malignant disease or other comorbidity with poor prognosis, viii) active infection or relevant inter-current illness, and ix) pregnancy (Table 1).

**Table 1:** Inclusion and exclusion criteria. CKD, chronic kidney disease; eGFR_CKD-EPI_, estimated glomerular filtration rate using the Chronic Kidney Disease Epidemiology Collaboration Equation

Inclusion criteria	Exclusion criteria
Age > 18 years	Kidney transplantation
	Myocardial infarction or unstable angina in the past three months and/or congestive heart failure class III-IV, according to the New York Heart Association criteria
	History of stroke, dementia, or other severe neurologic disorders Parkinson's disease, multiple sclerosis, etc.)
Patients with CKD at stages 2-4, i.e., with eGFR_CKD-EPI _<90 and ≥15 mL/min/1.73m^2^	Severe mental disorders
	Muscle disorders
	History of drug or alcohol abuse
Provision of informed written consent	Active malignant disease or any other comorbidity with poor prognosis
	Active infection or relevant inter-current illness
	Pregnancy

Data collection

**Figure 1:**
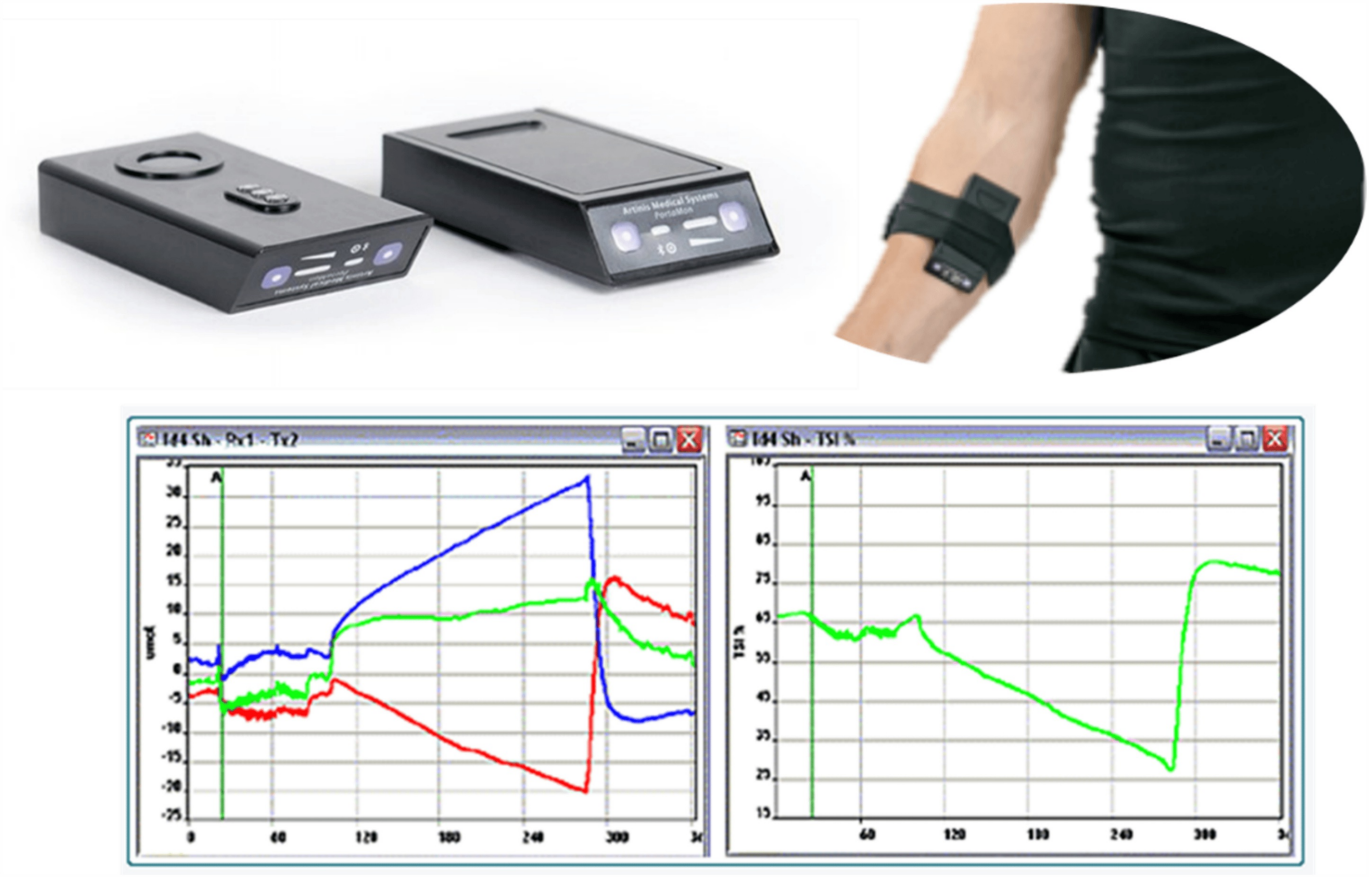
Muscle near-infrared spectroscopy setup.

Study participants were scheduled to visit our research unit after 12 hours of fasting and without using caffeine, alcohol, or tobacco; all participants were instructed to receive their standard morning medication. Baseline demographic and anthropometric characteristics, past medical history, and concomitant medication were recorded, and a physical examination was performed. Venous blood samples were collected for routine laboratory tests; all patients were also instructed to perform a 24-hour urine collection ending on the morning of evaluation to measure urinary protein, creatinine, sodium, and potassium levels. Office blood pressure measurements were performed in triplicate after 5-10 minutes of seated rest at the level of the brachial artery, with a 1- to 2-minute interval between each measurement, according to the recommendations [15]. Then, the cognitive function of all participants was assessed using the Mini-Mental State Examination (MMSE). Muscle and cerebral NIRS and microvascular reactivity were measured as described below (Figure 1). All procedures were performed in a quiet room with an ambient temperature of 23-24°C.

Definition of proteinuria

For the purposes of this analysis, we divided the population in CKD patients with and without proteinuria. Proteinuria was defined as urinary protein-to-creatinine ratio (UPCR) >200 mg/g, according to recommendations [16]. A blinded member of our team matched eligible patients with and without proteinuria in a 1:1 ratio for age, sex, and eGFR levels.

Assessments

NIRS is a non-invasive technique that provides reliable assessments of muscle and cerebral oxygenation and hemodynamics both at rest and during physical activity.

Muscle Oxygenation

Muscle oxygenation and microvascular reactivity were monitored via Portamon (Artinis Medical Systems, Elst, the Netherlands). NIRS continuously monitored the relative changes from baseline in oxygenated hemoglobin (O_2_Hb), deoxygenated hemoglobin (HHb), and total hemoglobin (tHb) in the microvasculature of the skeletal muscle. The tissue saturation index (TSI, %) was calculated as an absolute parameter of muscle oxygenation [17]. The device was placed on the participants’ forearm of the dominant hand to non-invasively monitor changes in muscle oxygenation [17].

During the measurements, the participants remained in the resting position for approximately 10 minutes, and arterial occlusion followed with their arm supported at the heart’s level. The cuff was inflated to supra-systolic levels (i.e., 250 mmHg) for 5 minutes (occlusion period) and then it was rapidly deflated for the start of the 3-minute reperfusion period. Stable blood flow/volume was verified by stable tHb. Μeasured variables of interest included the following: baseline TSI (at rest), minimum value of TSI obtained during occlusion (TSI_min_), and maximum value of TSI obtained during reperfusion (TSI_max_). The magnitude of TSI decline (TSI_ocl_ magnitude defined as the difference between baseline TSI and TSI_min_) and the TSI occlusion slope (TSI_ocl _slope) were calculated and used as indices of muscle’s oxidative capacity.

During reperfusion, the following indexes were assessed: magnitude of TSI increase during reperfusion (TSI_rep_ magnitude defined as the difference between TSI_max_ and TSI_min_) and the TSI reperfusion slope (slope of initial 30 seconds and slope to TSI_max_), which reflect the muscle’s capacity for oxygen delivery to demand, and the hyperemic response (difference between TSI_max_ and baseline TSI). The latter is an index of skeletal muscle microvascular reactivity that shows the ability of microvasculature to accommodate the increase in blood flow in response to a physiologic stimulus [17].

Following a 5-minute seated rest, the exercise protocol was performed. The participants’ maximal voluntary contraction (MVC) was measured with three trials (a 90-second rest between each attempt) (K-Force, K-Invent, Montpellier, France); the highest of the three readings was considered the patient’s MVC. Subsequently, the participant performed the 3-min submaximal intermittent exercise protocol (six sets of 30-second exercise at 35% MVC with a 3-second rest between sets), during which they had visual feedback to maintain force output to the predetermined percentage of the MVC. During handgrip, participants were advised to avoid the Valsalva maneuver and keep non-participating muscles relaxed. NIRS-derived variables of interest included the following: i) baseline TSI, ii) average value of TSI obtained during handgrip exercise (TSI_HG_), iii) minimum value of TSI obtained during handgrip exercise (TSI_HGmin_), and iv) average decline of TSI obtained during handgrip exercise (difference between baseline and TSI_HG_), which reflects the skeletal muscle oxygenation during exercise [17].

Cerebral Oxygenation

Cerebral oxygenation was monitored with Oxymon (Artinis Medical Systems Elst, The Netherlands), which detects relative changes in O_2_Hb, HHb, and tHb [18]. The NIRS sensor was placed over the prefrontal cortex, contralateral to the dominant hand. The O_2_Hb and hemoglobin difference (Hb_diff_, defined as the difference between O_2_Hb and HHb) were used as indices of tissue oxygenation, the HHb was used as an index of oxygen extraction capacity, and the tHb was used as an estimate of changes in regional tissue-blood volume [18].

Following a 5-minute seated rest, the exercise protocol was performed. The participants’ MVC was measured as the highest of three trials (90-second rest between each attempt) (K-Force, K-Invent). Subsequently, the participants performed the 3-minute submaximal intermittent handgrip exercise (six sets of 30-second exercise at 35% MVC with a 3-second rest between sets), during which they had visual feedback to maintain force output to the predetermined percentage of the MVC.

Statistical analysis

Statistical analysis was conducted using the Statistical Package for the Social Sciences (SPSS) Version 22 (IBM Corp., Armonk, NY). Continuous variables were expressed as mean ± standard deviation (mean±SD) or median (interquartile range) according to the normality of distribution assessed by the Shapiro-Wilk test. Categorical variables are presented as absolute frequencies and percentages (n, %). Between-group comparisons for continuous variables were performed using the independent samples t-test or the Mann-Whitney U test, as appropriate. The chi-square or Fisher’s exact test was used for comparisons of categorical variables. For all analyses, a p-value of <0.05 (two-tailed) was considered statistically significant.

## Results

Demographic and clinical characteristics

A total of 56 patients with pre-dialysis CKD of stages 2-4 were included and stratified by the presence or absence of proteinuria (28 proteinuric, 28 non-proteinuric). Table 2 depicts the demographic, clinical, and laboratory characteristics between groups. No differences were observed between the two groups in terms of age, sex, eGFR, and body mass index. Furthermore, no between-group differences in any major comorbidities were detected, while the use of major antihypertensive medications did not differ as well. No differences in MMSE score and handgrip MVC were observed.

**Table 2:** Baseline characteristics of proteinuric and non-proteinuric CKD patients. Continuous variables are reported as mean±standard deviation (mean±SD) or median (interquartile range) based on the normality of distribution tested with the Shapiro-Wilk test. Categorical variables are presented as absolute frequencies and percentages (n, %). Between-group comparisons for categorical variables (i.e., male gender, major comorbidities, antihypertensive agents) were made using the chi-square test and for non-normally distributed continuous variables (i.e., UPCR) with the Mann-Whitney U test. The rest of the comparisons for normally distributed variables are performed by applying the independent Student’s t-test. For all comparisons, a value of p<0.05 is deemed as statistically significant Symbols (for chi-square test statistics): ^a^degrees of freedom (df) = 1, phi coefficient (φ) = 0.00; ^b^df = 1, φ = 0.194 (small to medium effect size); ^c^df = 1, φ = 0.047 (small effect size); ^d^df = 1, φ = 0.120 (small effect size); ^e^df = 1, φ = 0.077 (small effect size); ^f^df = 1, φ = 0.082 (small effect size); ^g^df = 1, φ = 0.078 (small effect size); ^h^df = 1, φ = 0.098 (small effect size); ^i^df = 1, φ = 0.081 (small effect size); ^j^df = 1, φ = 0.154 (small effect size); ^k^df = 1, φ = 0.274 (small to medium effect size). ACEi, angiotensin-converting enzyme inhibitor; ARB, angiotensin II receptor blocker; BMI, body mass index; CAD, coronary artery disease; CCB, calcium-channel blocker; CKD, chronic kidney disease; eGFR, estimated glomerular filtration rate; MVC, maximal voluntary contraction; MMSE mini-mental state examination; UPCR, urinary protein-to-creatinine ratio

Parameter	Proteinuric CKD patients	Non-proteinuric CKD patients	p-value
N	28	28	-
Age (years)	69.6±10.4	67.3±11	0.428
Males (n, %)^a^	20 (71.4%)	20 (71.4%)	1.000
eGFR	39.5±19.1	44.1±10.2	0.271
BMI (kg/m^2^)	28.1±4.6	28.7±6.3	0.744
Major comorbidities			
Diabetes (n, %)^b^	12 (42.9%)	17 (60.7%)	0.264
CAD (n, %)^c^	5 (17.9%)	6 (21.4%)	1.000
Heart failure (n, %)^d^	2 (7.1%)	4 (14.3%)	0.668
Dyslipidemia (n, %)^e^	15 (53.6%)	13 (46.4%)	0.781
Hypertension (n, %)^f^	25 (89.3%)	24 (85.7%)	1.000
Antihypertensive medication			
ACEIs/ARBs (n, %)^g^	14 (50%)	16 (57.1%)	0.779
CCBs (n, %)^h^	22 (78.6%)	20 (71.4%)	0.726
Diuretics (n, %)^i^	8 (28.6%)	10 (35.7%)	0.771
β-blockers (n, %)^j^	11 (39.3%)	15 (53.6%)	0.406
α-blockers (n, %)^k^	9 (32.1%)	3 (10.7%)	0.097
Laboratory values			
Hemoglobin (g/dL)	12.9±1.8	13.5±1.4	0.188
Hematocrit (%)	39.6±5.3	40.9±4.1	0.334
Urea (mg/dL)	74.9±37.3	58.3±23.6	0.052
Creatinine (mg/dL)	1.98±0.9	1.57±0.34	0.030
Sodium (mmol/L)	139.4±2.4	138.4±3.0	0.154
Potassium (mmol/L)	4.9±0.4	4.7±0.4	0.047
Calcium (mg/dL)	9.5±0.7	9.9±0.5	0.032
Phosphate (mg/dL)	3.5±0.7	3.4±0.4	0.357
UPCR (mg/g)	672 (1431)	68 (52)	<0.001
MMSE (total)	28.1±1.4	27.2±2.0	0.305
Max MVC	22.0±7.6	24.1±7.6	0.311

Muscle NIRS

Table 3 displays muscle oxygenation parameters at rest, during occlusion-reperfusion, and during handgrip exercise. There were no significant differences between groups in resting TSI between the two groups (62.8±3.5 vs 62.4±3.1, p=0.679). Similarly, during occlusion, TSI_min_, TSI_ocl_ magnitude, and TSI_ocl_ slope were not different between proteinuric and non-proteinuric CKD patients, suggesting comparable resting muscle oxygenation and oxidative capacity. During reperfusion, proteinuric patients showed a trend for reduced hyperemic response (although this was not statistically significant) compared to non-proteinuric patients (7.3 ± 4.0 vs. 8.5 ± 4.5, p=0.296). Similarly, TSI recovery slopes (e.g., TSI_rep_ 10-second slope: 1.36 ± 0.69 vs. 1.67 ± 0.83, p=0.143) and the TSI slope to maximum (1.04 ± 0.49 vs. 1.26 ± 0.70, p=0.176) were numerically lower in the proteinuric group, indicating a trend toward impaired microvascular reactivity. Finally, during the handgrip exercise protocol, there were no significant differences in TSI_^HG^_, TSI_HGmin_, or average TSI decline between groups (Table 3).

**Table 3:** Muscle NIRS parameters during occlusion-reperfusion and handgrip exercise in proteinuric and non-proteinuric CKD patients. All continuous variables are normally distributed (tested with the Shapiro-Wilk test) and reported as mean±standard deviation (mean±SD). Between-group comparisons are made using the independent Student’s t-test. For all comparisons, a value of p<0.05 is deemed as statistically significant. CKD, chronic kidney disease; NIRS, near-infrared spectroscopy; max, maximum; TSI, tissue saturation index; TSI_HG_, TSI during handgrip exercise; TSI_HGmin_, minimum value of TSI during handgrip exercise; TSI_max_, maximum value of TSI during reperfusion; TSImin, minimum value of TSI during occlusion; TSI_ocl_, TSI during occlusion; TSI_rep_, TSI during reperfusion

Parameter	Proteinuric CKD patients	Non-proteinuric CKD patients	t-value	p-value
Baseline TSI	62.8±3.5	62.4±3.1	0.416	0.679
Occlusion				
TSI_min_	36.6±9.4	35.4±13.5	0.377	0.708
TSI_ocl_ magnitude	26.3±8.8	27.1±11.7	-0.288	0.774
TSI_ocl_ slope	-0.09±0.04	-0.09±0.05	0.447	0.657
Reperfusion				
TSI_max_	70.0±4.8	70.9±4.6	-0.663	0.510
TSI_rep_ magnitude	33.5±11.3	35.6±14.8	-0.582	0.563
10-second TSI_rep_ slope	1.36±0.69	1.67±0.83	-1.485	0.143
20-second TSI_rep_ slope	1.40±0.65	1.62±0.74	-1.191	0.239
30-second TSI_rep_ slope	1.18±0.52	1.30±0.59	-0.806	0.424
TSI_rep_ slope to max	1.04±0.49	1.26±0.70	-1.373	0.176
Hyperemic response	7.3±4.0	8.5±4.5	-1.056	0.296
Handgrip exercise				
TSI_HG_	51.5±6.4	53.4±8.3	-0.977	0.333
TSI_HGmin_	44.6±8.6	45.7±10.5	-0.438	0.663
TSI average decline	11.2±6.2	10.2±6.1	0.659	0.513

Cerebral NIRS

Table 4 shows the average responses in cerebral NIRS parameters during exercise for both groups. As shown, the average increases observed during exercise in O_2_Hb were significantly lower in proteinuric compared to non-proteinuric patients (0.92±0.78 vs 1.49±0.86, p=0.012). Similarly, the average tHb response as well as Hb_diff_ levels were significantly lower in proteinuric patients (tHb, 0.43±0.98 vs. 1.00±0.85, p=0.023; Hb_diff_: 1.41±0.96 vs 1.98±1.12, p=0.044). However, no between-groups differences were detected for HHb (p=0.961). 

**Table 4:** Average response in cerebral NIRS parameters during handgrip exercise between proteinuric and non-proteinuric CKD patients. All average responses of cerebral NIRS parameters are normally distributed (tested with the Shapiro-Wilk test) and reported as mean±standard deviation (mean±SD). Between-group comparisons are made using the independent Student’s t-test. For all comparisons, a value of p<0.05 is deemed as statistically significant. CKD, chronic kidney disease; Hb_diff_, hemoglobin difference; HHb, deoxygenated hemoglobin; NIRS, near-infrared spectroscopy; O_2_Hb, oxygenated hemoglobin; tHb, total hemoglobin

Parameter	Proteinuric CKD patients	Non-proteinuric CKD patients	t-value	p-value
O_2_Hb (μmol/L)	0.92±0.78	1.49±0.86	-2.601	0.012
HHb (μmol/L)	-0.49±0.59	-0.48±0.51	-0.049	0.961
tHb (μmol/L)	0.43±0.98	1.00±0.85	-2.341	0.023
Hb_diff_ (μmol/L)	1.41±0.96	1.98±1.12	-2.060	0.044

## Discussion

This post-hoc, exploratory analysis investigates, for the first time, the effects of proteinuria on skeletal muscle and cerebral oxygenation, as well as on muscle microvascular reactivity in patients with pre-dialysis CKD. In the analysis for muscle oxygenation, no between-group differences were observed in muscle oxygenation at rest, during occlusion, and during handgrip exercise. However, during reperfusion, proteinuric CKD patients exhibited a trend toward a numerically slower increase in TSI (lower TSI slope) and a numerically lower hyperemic response, suggesting more impaired microvascular reactivity. With regard to cerebral oxygenation, the average responses in O_2_Hb and Hb_diff_ during handgrip (indices of tissue oxygenation) were significantly lower in proteinuric patients. A similar course was detected for the average tHb response, an index of changes in regional tissue blood volume. No significant differences in HHb response between groups were found, suggesting no differences in oxygen extraction capacity.

Endothelial dysfunction, characterized by an imbalance between agents with vasodilating, antimitogenic, and antithrombogenic properties (endothelium-derived relaxing factors) and agents with vasoconstricting, prothrombotic, and proliferative properties (endothelium-derived contracting factors), is associated with adverse outcomes in patients with CKD [19]. It occurs early in CKD and shows a progressive deterioration with advancing CKD [6,7]. In parallel, it has been associated with target-organ damage, progression of CKD, cardiovascular events, and mortality in these patients [6,8,9]. Proteinuria, a key marker of glomerular injury, is also strongly and independently linked to endothelial dysfunction [12].

Over the years, a variety of techniques have been employed to assess endothelial function in humans, including functional methods, such as venous occlusion plethysmography (VOP), flow-mediated dilatation (FMD), laser-Doppler flowmetry, laser-speckle contrast imaging (LASCA), and nailfold capillaroscopy, as well as circulating biomarkers, such as asymmetric dimethylarginine (ADMA), endothelial microparticles, and cell adhesion molecules [13]. NIRS is a non-invasive tool assessing local tissue oxygenation that allows evaluation of microvascular reactivity in skeletal muscles [17]. The NIRS device operates by emitting near-infrared light at wavelengths that are differentially absorbed by O_2_Hb and HHb and calculates the tissue oxygen saturation index. By applying an arterial occlusion/reperfusion maneuver, NIRS enables the assessment of skeletal muscle oxidative capacity and microvascular reactivity, particularly the evaluation of downstream hyperemia, which cannot be captured by more traditional macrovascular methods [17]. Additionally, NIRS permits dynamic monitoring of skeletal muscle oxygenation during exercise, offering valuable insight into the physiological balance between oxygen delivery and utilization. With regard to cerebral oxygenation, NIRS non-invasively monitors alterations and assesses relative changes from baseline for oxygenated, deoxygenated, and total hemoglobin [17].

Very few studies have examined muscle oxygenation and skeletal muscle microvascular function with NIRS in CKD [19]. Resting muscle oxygenation was significantly lower in hemodialysis patients than healthy controls [20,21] and associated with hemoglobin, serum phosphate, and albumin levels [20,21]. In a previous study evaluating muscle oxygenation during a shuttle-walk test in 24 patients with pre-dialysis CKD, slower muscle oxygenation recovery kinetics post-exercise was observed [22]. More recently, we showed that the microvascular hyperemic response in skeletal muscles was significantly impaired in CKD and deteriorated toward more advanced CKD stages, while muscle oxygenation during exercise also tended to be worse in CKD patients than controls [7]. In the present analysis, our muscle data similarly reflect a subtle, yet functionally relevant, impairment in proteinuric CKD. The blunted TSI recovery slopes and hyperemic responses during reperfusion suggest that proteinuria may hinder both oxygen delivery and the microvasculature’s ability to dynamically respond to demand. These differences were not statistically significant, probably due to the small sample size, but they are consistent with prior work documenting progressive microvascular rarefaction and reduced capillary recruitment with advancing CKD stages (~0.8-1% difference in hyperemic response between each CKD stage) [7].

Studies assessing cerebral oxygenation with NIRS in CKD are also limited. Prior studies in end-stage kidney disease reported lower resting cerebral oxygenation in hemodialysis patients compared to healthy individuals [23,24]. Low cerebral oxygenation was correlated to worse cognitive performance in these patients [25]. In addition, in a small study in 12 hemodialysis patients and 12 controls, hemodialysis patients displayed lower VO2 peak and blunted muscular deoxyhemoglobin increase during exercise, suggesting an increase in oxygen affinity and/or mitochondrial dysfunction in this population. In non-dialysis CKD, resting cerebral oxygenation was strongly and positively correlated with eGFR levels [26]. Moreover, we have recently shown that there is a blunted increase in cerebral oxygenation and regional blood volume during exercise with advancing CKD stages [14]. In the current analysis, although both proteinuric and non-proteinuric groups had similar eGFR levels, the significantly lower O_2_Hb and tHb responses in the proteinuric group suggest that proteinuria may be an additional, independent factor that compromises cerebral hemodynamics. This aligns with prior studies that used magnetic resonance imaging and linked proteinuria to impaired cerebral perfusion [27,28]. From a pathophysiological point of view, a plausible mechanism is that proteinuria reflects heightened systemic endothelial dysfunction, leading to impaired vasodilatory responses. Proteinuric patients have elevated levels of circulating ADMA and other markers of nitric oxide synthase inhibition, which can compromise vascular tone regulation across multiple circulatory beds [9]. Another contributing factor may be structural-microvascular rarefaction due to chronic inflammation and oxidative stress, both of which are aggravated by proteinuria [12]. Impaired cerebrovascular reactivity due to increased atherosclerosis and stiffness of the larger brain vessels could also play a role, as both atherosclerosis and arteriosclerosis are closely associated with cerebral perfusion and oxygenation [18,29] and deteriorate with proteinuria [30].

The present study has several strengths. It is the first study to investigate the effects of proteinuria on muscle and cerebral oxygenation with the use of real-time measurements via NIRS at rest, during exercise, and after an occlusion-reperfusion maneuver. In parallel, it followed a strict protocol, including an elaborate matching for age, sex, and eGFR. However, several limitations warrant consideration. First, a formal power calculation was not performed, as this was a post-hoc, exploratory analysis of an existing dataset. Consequently, the study may be underpowered to detect small or moderate differences in tissue oxygenation or microvascular reactivity between groups. As such, our findings should be interpreted as hypothesis - generating rather than conclusive. Second, the modest sample size may have precluded the detection of subtle between-group differences in muscle responses. Third, as cerebral NIRS focuses only on prefrontal cortex oxygenation, other regions critical to motor and cognitive control were not assessed in this study; future studies assessing other cerebral regions are encouraged to shed more light on this field. Fourth, we did not include supplementary biomarker measurements (e.g., inflammatory or endothelial markers) to further elucidate underlying mechanisms. Finally, participants were recruited from a single tertiary nephrology center and underwent strict eligibility screening (Table 1) and matching for age, sex, and eGFR. While this careful matching enhances internal validity, it may limit the external validity of our findings. The study population may not fully reflect the broader CKD population, i.e., patients with earlier stages or those with kidney failure.

## Conclusions

In conclusion, our findings suggest a potential association between proteinuria in CKD and altered cerebral oxygenation responses during physical stress, as well as possible impairments in peripheral microvascular reactivity. These preliminary observations warrant further investigation in larger, prospective studies to better understand whether and how such microvascular alterations may relate to functional capacity, QoL, and clinical outcomes in this population.
